# Epidemiologic, Imaging, and Clinical Issues in Bezold’s Abscess: A Systematic Review

**DOI:** 10.3390/tomography8020074

**Published:** 2022-04-01

**Authors:** Silvia Valeggia, Matteo Minerva, Eva Muraro, Roberto Bovo, Gino Marioni, Renzo Manara, Davide Brotto

**Affiliations:** 1 Department of Medicine, Radiology Institute, University of Padova, Via Giustiniani 2, 35128 Padova, Italy; silvia.valeggia@studenti.unipd.it (S.V.); matteo.minerva@studenti.unipd.it (M.M.); 2 Camposampiero Hospital, 35012 Camposampiero, Italy; eva.muraro@aulss6.veneto.it; 3 Section of Otorhinolaryngology, Department of Neurosciences, University of Padova, Via Giustiniani 2, 35128 Padova, Italy; roberto.bovo@unipd.it (R.B.); davide.brotto@unipd.it (D.B.); 4 Neuroradiology Unit, Department of Neurosciences, University of Padova, Via Giustiniani 2, 35128 Padova, Italy; renzo.manara@unipd.it

**Keywords:** Bezold’s abscess, Bezold, abscess, cholesteatoma, MRI

## Abstract

Bezold’s abscess is a deep neck abscess related to otomastoiditis. Due to the insidious clinical presentation, diagnosis can be extremely challenging, leading to delays in treatment and possible life-threatening complications. The literature currently provides a fragmented picture, presenting only single or small number of cases. The present study aims at examining our experience and the literature findings (based on PRISMA criteria) of 97 patients with Bezold’s abscess, summarizing their epidemiology, pathogenesis, clinical presentation, imaging findings, and treatments. Bezold’s abscess is found at any age, with overt male prevalence among adults. The clinical presentation, as well as the causative pathogens, are strikingly heterogeneous. Otomastoiditis and cholesteatoma are major risk factors. A clinical history of otitis is commonly reported (43%). CT and MRI are the main diagnostic tools, proving the erosion of the mastoid tip in 53% of patients and the presence of a concomitant cholesteatoma in 40%. Intracranial vascular (24%) or infectious (9%) complications have also been reported. Diagnosis might be easily achieved when imaging (CT) is properly applied. MRI has a limited diagnostic role, but it might be crucial whenever intracranial complications or the coexistence of cholesteatoma are suspected, helping to develop proper treatment (prompt antibiotic therapy and surgery).

## 1. Introduction

**Figure 1:**
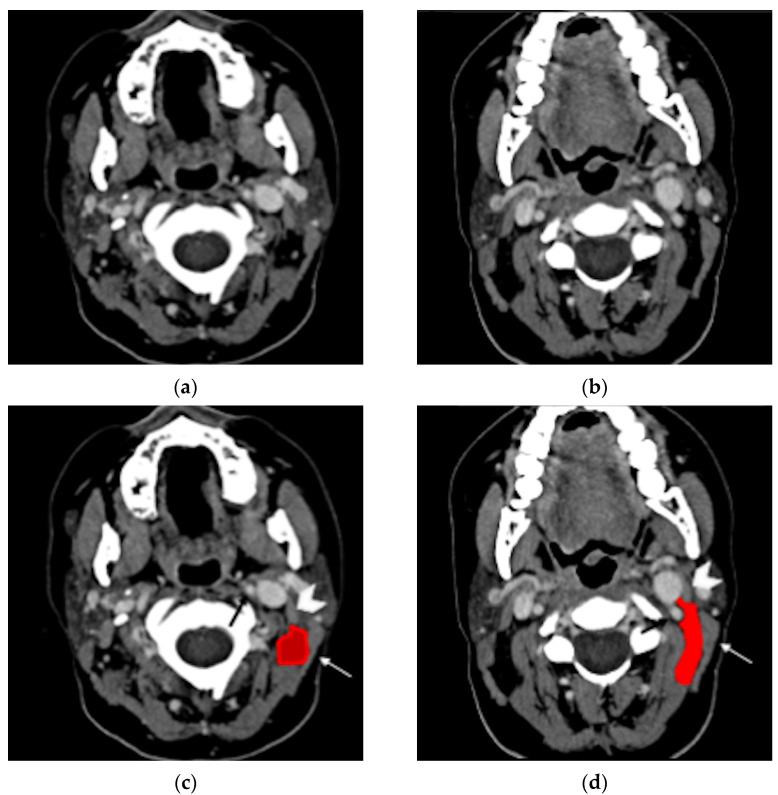
Normal anatomy, post-contrast axial CT (**a**,**b**). In the same images with annotations (**c**,**d**), the digastric muscle (white arrowhead) and the sternocleidomastoid muscle (white arrow) delimitate the posterior cervical space (red). The carotid artery can be identified nearby (black arrow).

Bezold’s abscess is a potentially life-threatening laterocervical/deep neck abscess complicating the course of an otomastoiditis [1,2]. First described in 1885 by Friedrich von Bezold [3], Bezold’s abscess is the spread of an aggressive middle ear and mastoid infectious process, through an erosion at the digastric ridge, deep in the neck between the digastric and sternocleidomastoid muscles (Figure 1).

Despite the high incidence of acute otitis media, Bezold’s abscesses are rare, especially in the pediatric population [4]. In a recent study considering around 5 million visits for acute otitis media, no patient presented such a complication [5]. However, Bezold’s abscesses are often underreported: over the last 20 years, at least four cases have been observed in the Neuroradiology Unit of Padova University Hospital. In addition, because of the high risk for life-threatening intracranial, neck, or even mediastinal complications [6], Bezold’s abscess requires early diagnosis, treatment, and surgery [7]. As the existing knowledge is based on single cases or small series of cases, providing a fragmented and heterogeneous picture of Bezold’s abscess, we have reviewed the existing literature focusing on epidemiology, pathogenesis, clinical presentation, imaging tools and features, differential diagnosis, and treatments.

## 2. Materials and Methods

**Figure 2:**
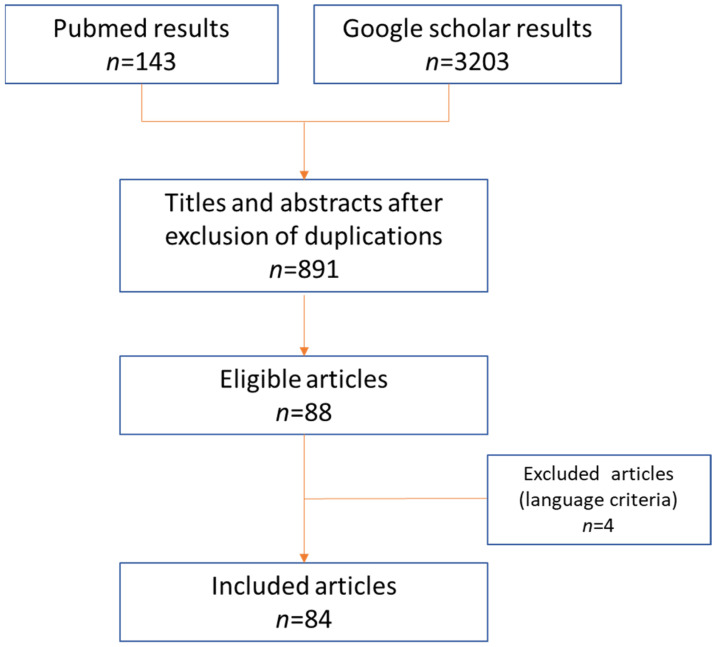
Literature search results according to PRISMA criteria.

The PubMed database and Google Scholar were screened up to August 2021, using the following Keywords and Meshes: “Bezold’s abscess”; “Bezold” AND “abscess”; “Bezold’s mastoiditis”. The literature search was performed by two authors independently (SV and MM), according to PRISMA criteria (Figure 2).

All the retrieved publications were evaluated to identify the most relevant articles. Duplications or aggregations of pre-existing data were excluded; only articles in English and Spanish were included. The reference lists of selected articles were also analyzed to identify additional studies.

Every misalignment the authors had with regards to article eligibility was solved through discussion.

Eighty-eight articles were identified: four were excluded due to unavailability of their full text both online and in main Italian medical libraries. Eighty-four studies were therefore considered; publication year ranged between 1898 and 2021.Our direct experience of four cases (three of them already published) were also considered for discussion and for imaging illustration.

The chi-square test and Student’s t-test were used when appropriate; a *p* < 0.05 was considered significant.

## 3. Results

A total of 97 patients with Bezold’s abscess was found (average age: 34.6 ± 22.3 years; range: 10 months to 87 years; mode: 35 years; median: 29 years). Males were substantially prevalent (74/97, 76%).

There was no significant difference in age between males and females (*p* = 0.4), whereas sex distribution differed significantly between pediatric and adult patients (*p* = 0.01). Bezold’s abscess occurred similarly in males and females who were up to 5 years old (four male vs. three female patients; male-to-female ratio = 1.33) and under 18 years old (13 male vs. 10 female patients; male-to-female ratio = 1.30). In contrast, among adult and elderly patients, males were strikingly more frequently affected (61 male vs. 13 female patients: male-to-female ratio = 4.69).

Microbiological cultures were commonly reported in Bezold’s abscesses (67/97, 69%), with heterogeneous results (Table 1).

Bezold’s abscess clinical onset was also highly heterogeneous (Table 2).

CT was performed in 80% of patients, MRI in 22%. Mastoid tip erosion was frequently documented (51/97 patients; 53%), especially when underlying cholesteatoma was present. Among the 35 patients with concomitant cholesteatoma, 28/35 had mastoid tip erosion (*p* < 0.001).

Two further subjects had a defect medial to the mastoid tip, one of them in presence of cholesteatoma, whereas 7/97 had an intact mastoid.

Intracranial involvement was heterogeneous and included infectious (abscess 2/97, empyema 5/97, meningitis 6/97) and vascular complications (venous sinus thrombosis 21/97, stroke 2/97).

## 4. Discussion

### 4.1. Epidemiology

Bezold’s abscess is rare but can be found at any age. Its occurrence in the first five years of life has been considered anecdotal, due to the lack of pneumatization of the mastoid cells and the consequently thicker mastoid walls [8] that hamper the spread of the infection through this path. However, children under five represent nearly 7% of reported patients; this could be explained by a mere publication bias due to the well-known rarity and atypical presentation in the first years of life or by higher frequency of mastoiditis in children.

Furthermore, sex differences in mastoid development and pneumatization [9,10] might likely favor the spread of suppurative processes in adult males, among whom the frequency of Bezold’s abscess is higher.

### 4.2. Pathogenesis

Bezold’s abscess typically complicates the course of chronic/recurrent otomastoiditis, even though few exceptions have been reported (acute otomastoiditis, external otitis, os tympanicum cholesteatoma). This specific type of abscess accounts for 6% of otogenic abscesses [11]. Indeed, otitis media commonly spreads to the mastoid. Subsequent inflammatory hypertrophy of the mucosa of aditus ad antrum might block the suppurative process into the mastoid cells, giving origin to coalescent mastoiditis [12].

According to the currently leading hypothesis, the retained purulent collection can lead to erosion of the cortical bone in a locus minoris resistentiae, the digastric groove, forcing the drainage of the pus into the neck (Bezold’s abscess) [3]. This hypothesis is supported by the common detection of cholesteatomas in Bezold’s abscess (40%).

Cholesteatomas are slowly growing masses usually appearing in the middle ear, occupying the Prussak space, expanding upwards and displacing the ossicles [13]. When a middle ear infection occurs, its growth pattern might block the path towards the external acoustic meatus [14] facilitating Bezold’s abscess formation. Notably, cholesteatoma may worsen the ventilation of the ear cavities promoting recurrent superinfections. In addition, bone erosion might be facilitated and is more conspicuous in the presence of cholesteatoma.

Among the 39 patients with cholesteatoma, only one showed an intact mastoid, as the lesion stemmed from the os tympanicum [15]. In some cases, the cholesteatoma might drop into the neck through Bezold’s abscess pathway [16,17]. Furthermore, around 28% (11/39) of patients with Bezold’s and cholesteatoma had a previous history of oto-mastoid surgery. In these patients, the risk of Bezold’s abscess might be further increased by the presence of post-surgery bone alterations, ref. [8] thus justifying the use of follow-up MRI to promptly detect cholesteatomatous residuals or recurrences (see Section 4.7).

Microbiological cultures’ results were strikingly heterogeneous in the reviewed cases, possibly also because early empirical antibiotics therapy might hamper pathogen identification. The large spectrum of pathogens emerging from the analysis of existing literature has obvious implications for diagnosis and treatment.

### 4.3. Clinical Presentation

Bezold’s abscess might be difficult to recognize, as its clinical presentation is highly heterogeneous, ranging from signs of neck tissue inflammation or fever to otorrhea or facial paralysis. Neck symptoms are the most reported (Table 2), even though publication bias cannot be excluded.

The evaluation through palpation is limited, as the purulent collection lies deep in the neck [7,8], where muscular and fascial planes constitute an anatomic barrier to the spread of the pus towards the surface.

However, involvement of the neck’s subcutaneous layers or skin is not rare in the literature (11/97 cases [8,18,19,20,21,22,23,24,25,26,27]), showing that the anatomical boundaries might be sometimes overcome, leading to unexpected diffusion pathways.

Bezold’s abscess is a consequence of a mastoiditis [3,8] accompanied by an history of acute (23%), chronic (19%), or recurrent (2%) otitis media; in five patients, a concomitant otitis externa was present [14,28,29,30]. Therefore, history of otomastoiditis and ipsilateral cervical swelling should raise the suspicion of Bezold’s abscess and proper radiological examinations should be applied. Notably, in the pediatric population, acute otitis media might show an asymptomatic or paucisymptomatic course, even without the presence of otorrhea [31,32].

Patient history, imaging, or surgical inspection usually reveal the presence of cholesteatoma, although in a few cases (10/39 cases) its existence has been detected by simple otoscopy [19,23,25,33,34,35,36,37,38,39]. In patients with neck pain or swelling, otoscopic evaluation is of utmost importance because it might show signs of otitis, raising suspicion of Bezold’s abscess (Table 2).

Laboratory parameters are usually altered, with elevated leukocytes and C-reactive protein or erythrocyte sedimentation rate, consistently with a bacterial infection. In rare cases (four patients in literature) laboratory tests may be unremarkable or slightly above normality. In these cases, tuberculous otitis media [25,40], poorly controlled HIV [41], and cholesteatoma [42] were the underlying diseases and fever was absent [41,42] or not reported [25,40].

### 4.4. Differential Diagnosis

Differential diagnosis mainly includes lymphadenopathies, infected branchial cysts, temporal bone subperiosteal empyema, and other neck abscesses. The differential diagnoses are derived from available case reports investigating the main reasons for Bezold’s abscess diagnostic delay.

Bezold’s abscess might mimic a lymphadenopathy accompanying otitis [43]. Neck ultrasonography might help with recognizing reactive lymph nodes or identifying hypo/anechoic collections [44]. Infected branchial cysts might have a clinical and radiological presentation similar to Bezold’s abscess, but the mastoid is not involved, and bone erosion is never observed.

**Figure 3:**
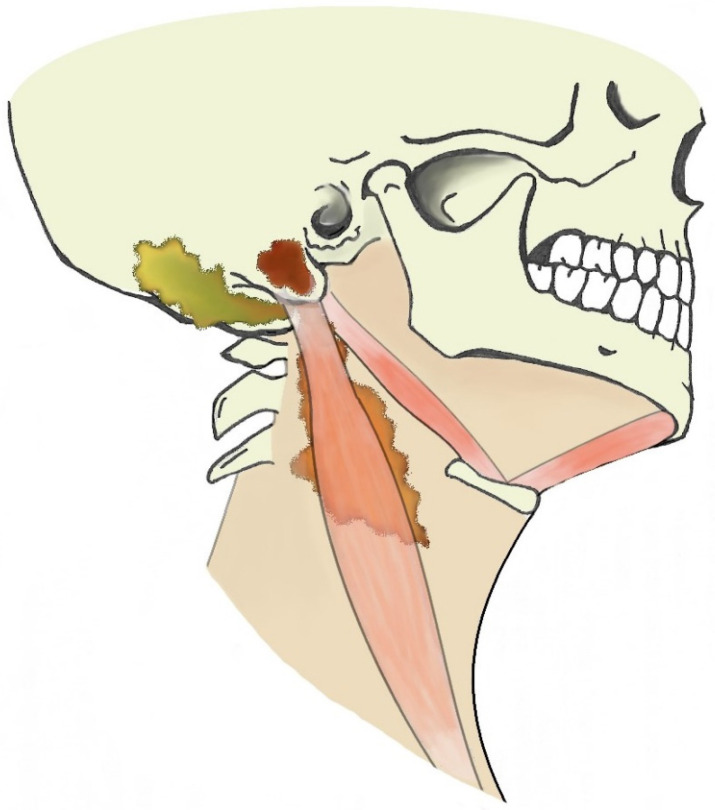
Illustration of extracranial otogenic abscesses. Bezold’s abscess (orange), Citelli’s abscess (yellow), subperiosteal empyema (brown).

When dealing with suppurative processes originating from the mastoid bone, the diffusion pathway through the neck should be considered as this might be pivotal for subsequent evolution and for treatment planning. Suppurative processes from the mastoid might spread through bone erosion along three different paths: medial, lateral, or posteromedial [45]. Due to the complex anatomy of the several neck muscles and fasciae connected to the mastoid, the site of bone erosion will determine the subsequent diffusion path of the suppurative process that is eventually classified with different eponyms (Figure 3).

A bone erosion medial to the mastoid allows the pus to spread to the posterior cervical and perivertebral spaces, deep into the sternocleidomastoid muscle (Bezold’s abscess); a posteromedial bone erosion can result in pus spreading posteriorly to the insertion of the digastric muscle and in the occipital region (Citelli’s abscess); a bone erosion lateral to the mastoid can give origin to a subperiosteal empyema that usually reaches the surface in the peri-mastoid subcutaneous spaces. However, this strict classification might not be appropriate in case of neck anatomy variants, erroneous identification of suppurative diffusion processes or coexistence of multiple paths. In fact, literature reports cases of Bezold’s abscess concomitant to subperiosteal empyema [22,30,38,46,47,48,49], occurring after mastoid surgery (i.e., consistent with a postoperative complication [24]) or spreading into the occipital region [50,51]. A spread to retro-parapharyngeal spaces has also been described in literature [8,32,34,42,52].

### 4.5. Imaging Features

Ultrasonography can represent a valuable first-line diagnostic tool, especially with children, to exclude reactive lymph nodes and detect neck abscesses that appear as anechoic or hypoechoic inhomogeneous collections [44].

However, CT and MRI are the best tools in identifying abscesses and concomitant mastoiditis features and providing useful information for the surgical approach as simple neck abscess drainage might be insufficient for eradicating the infection and ultrasonography shows only the “tip of the iceberg” and not the source of infection [8]. In addition, a precise differentiation between Bezold’s abscess and other neck abscesses of otogenic origin might help the surgeon with localizing the bone defect while evaluating the mastoid [45].

### 4.6. CT

**Figure 4:**
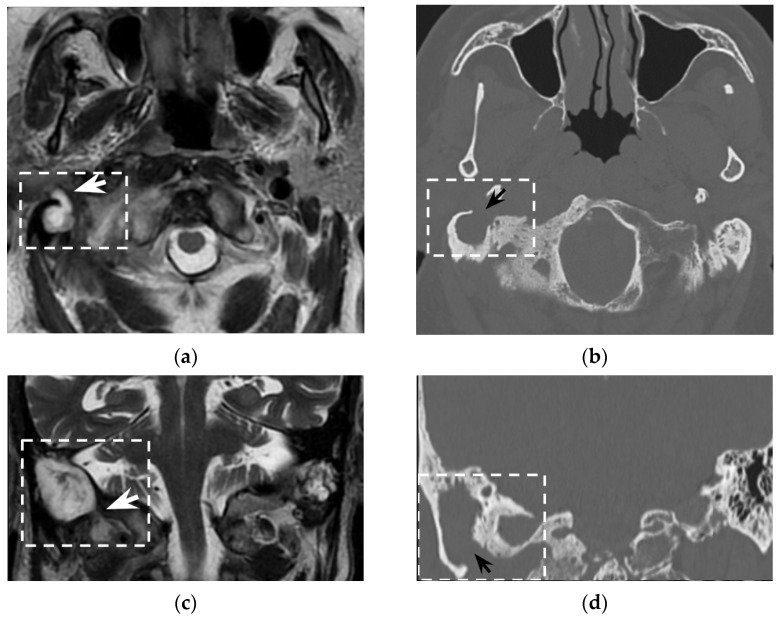
Eighty-seven-year-old man with history of external mycotic otitis. T2w MRI in axial (**a**) and coronal (**c**) planes show abnormal right mastoid (dotted rectangle) filled with hyperintense material that spreads into the neck (white arrows). Axial (**b**) and coronal (**d**) bone CT images show opacified right mastoid, absence of bone trabeculae, and a wide defect (black arrows) at the mastoid tip. Images were consistent with an infected cholesteatoma eroding the mastoid tip and spreading into the neck. The finding was confirmed at surgery.

In the clinical suspicion of a Bezold’s abscess, temporal bone CT and contrast enhanced neck CT are the gold standard [7] for defining two main diagnostic aspects: (1) the site of mastoid bone erosion in the context of a mastoiditis and (2) the anatomical boundaries of the neck suppurative collection. Temporal bone CT typically shows signs of mastoiditis, such as opacification of the mastoid cells and erosion of the mastoid bone trabeculae. High resolution (slice thickness < 1 mm), coronal plane reconstructions, high frequency bone kernel and bone window are suggested to enable the detection of small bone interruptions at the mastoid tip, generally at the digastric groove (Figure 4).

**Figure 5:**
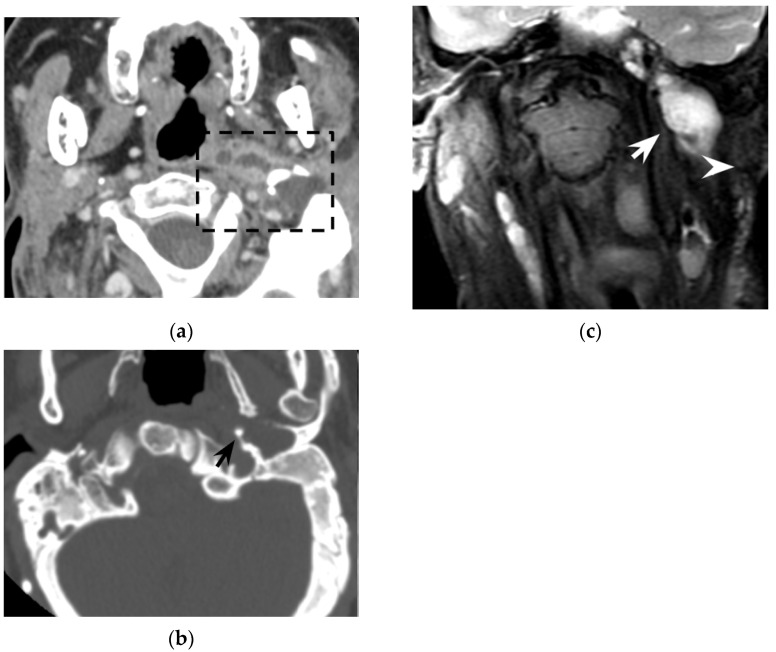
Thirty-year-old woman with oculo-auricular-vertebral spectrum. Contrast-enhanced axial CT (**a**) shows hypodense left neck abscess in the parapharyngeal and pharyngeal mucosal spaces, with minor involvement of the visceral and posterior cervical space (dotted rectangle). Note the peripheral enhancement and the soft tissue oedema. Axial CT with bone kernel (**b**) shows hypodense cholesteatoma eroding the os tympanicum (black arrow) as the origin of the neck abscess. Coronal T2w MRI (**c**) shows an hyperintense neck collection (white arrow) medial to the sternocleidomastoid muscle (white arrowhead). The patient proved to have a Bezold’s abscess associated with an os tympanicum cholesteatoma.

However, despite its crucial pathogenic role for the suppurative path, bone defects in the mastoid tip have been precisely outlined only in around half of all literature reports (51/97 patients, 53%). Indeed, in 7/97 patients (7%) the mastoid tip was considered intact, revealing that the diagnosis of Bezold’s abscess can be achieved even without overt bone erosion, especially in younger children (3/7 patients were younger than 5 years old). The incomplete pneumatization of mastoid cells before the age of five is well known and is supposed to hinder the diffusion of the suppurative process across the thickened mastoid bone wall. Before the age of five, more destructive infectious processes or emissary vein bone canals likely allow Bezold’s abscess occurrence [8]. A recent case of a woman affected by Goldenhar syndrome with an unremarkable mastoid bone (Figure 5) highlighted those atypical origins of Bezold’s abscess that should be accurately investigated in syndromic patients [15].

Temporal bone CT also allows for detection of intracranial bone erosions possibly leading to further life-threatening complications [8]: sinus thrombosis, posterior cranial fossa abscess/empyema, or meningitis/meningoencephalitis have been reported in 25/97 patients.

Contrast-enhanced neck CT aims at demonstrating and precisely defining the anatomical limits of the neck abscess that appears as a low-attenuation collection with typical rim enhancement (Figure 4) and surrounding soft tissue oedema. Neck CT should be reconstructed with 2–3 mm thick slices and a soft tissue kernel and window. The suppurative collection is usually located under the sternocleidomastoid muscle, in the posterior-cervical or perivertebral spaces, but extensions into the retropharyngeal space [30,51], parapharyngeal space [30,42,45,53], or even into the thorax [24,26,46,54] have been described, coherently with neck fascial anatomy and least resistance pathways.

In some patients the abscess might trespass the deep cervical fascia and reach the surface (see Section 4.3) subverting neck anatomy and escaping the classic path of Bezold’s abscess diffusion. For this reason, even the precise characterization of mastoid tip bone destruction might not accurately predict the path of infection. Contrast-enhanced CT might be extended to the brain if neurological conditions are rapidly deteriorating; CT angiography can be applied to rapidly diagnose dural sinus thrombosis. However, MRI should be preferred whenever intracranial complications are suspected [8].

### 4.7. MRI

The role of MRI in the diagnosis of Bezold’s abscess is generally limited, being a “problem solving modality" used when intracranial complications or osseous disease are expected. In literature, MRI findings are reported for only 21/97 patients. Indeed, the first MRI report dates to 2001. The low rate of MRI reports likely depends on two concomitant factors: MRI was not available at diagnosis and CT was considered sufficient for proper surgical planning and patient management. However, MRI might be better in specific clinical circumstances (for example, when intracranial complications are present, a cholesteatoma is suspected, the purulent content of intracranial or neck collections should be proven) as diffusion-weighted imaging (DWI) best differentiates non-infectious fluid collections from bacterial abscesses.

Bezold’s abscess entails a moderate risk of intracranial vascular (23/97) or further infectious (9/97) complications. MRI protocols should therefore include DWI and vascular imaging. DWI best recognizes suppurative collections (abscess, empyema, or even intraventricular debris) showing hyperintense signal on DWI with usually decreased apparent diffusion coefficient values (i.e., restriction of water molecule diffusion). DWI also detects cytotoxic oedema and intravascular thrombi helping in recognizing recent ischemic strokes or sinus venous thrombosis. Artery and venous intracranial MR-angiography can detect vessel occlusion consistent with thrombosis, thus confirming CT-angiography findings and allowing a less invasive follow-up. These sole sequences cover most intracranial complications of mastoiditis associated with Bezold’s abscess and are therefore of utmost importance for subsequent patient management.

**Figure 6:**
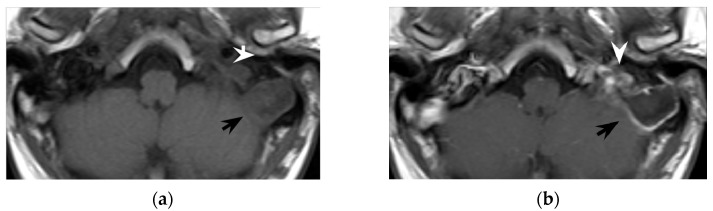

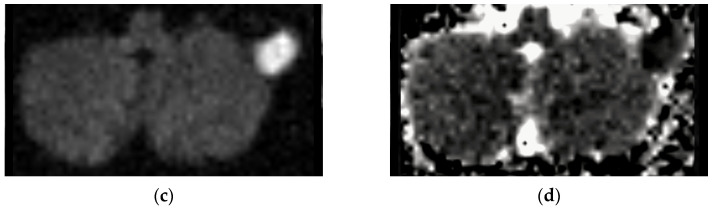
Thirty-seven-year-old man with chronic otitis media and history of ear surgery. MRI T1w (**a**) and contrast-enhanced T1w (**b**) sequences showing hypo-isointense material in the left mastoid (black arrows) spreading in the neck, in the posterior cervical space (white arrowheads). Note the mastoid mucosal enhancement, suggestive for mastoiditis, and the peripheral enhancement of the neck component (**b**). The material in the mastoid is hyperintense in diffusion-weighted imaging (**c**) and shows inhomogeneous apparent diffusion coefficient values (**d**), consistent with purulent collection and cholesteatoma. Bezold’s abscess and left mastoid cholesteatoma were confirmed at surgery.

In the literature, cholesteatoma ipsilateral to Bezold’s abscess was reported in around 40% of patients. Cholesteatoma likely favors repeated mastoid bacterial superinfections and facilitates, by bone erosion, the spread of suppurative process into the neck (see Section 4.2). In several patients, cholesteatoma was diagnosed at surgery (11/97) and in a few cases it was partially an unexpected finding. However, MRI might easily recognize cholesteatoma preoperatively as these cysts appear as strikingly hyperintense DWI ovoid masses [42]. Differently from purulent collections, apparent diffusion coefficient (ADC) values might be slightly increased in cholesteatomas, suggesting a T2 shine-through effect more than a true water diffusion restriction [55]. From a technical point of view, whereas investigating the mastoid and the upper neck, non-echo-planar (non-EPI) DWI sequences should be preferred to minimize mastoid air/bone susceptibility artifacts that could mask the cholesteatoma [56]. DWI represents a very powerful tool also in postoperative and follow-up examinations, allowing for the detection of small cholesteatomatous residuals or recurrences (Figure 6), especially those with a diameter larger than 2–4 mm [57].

As far as neck evaluation, MRI usually shows a well-defined area of low T1 and high T2 signal with DWI restriction, a peripherally enhancing rim, and surrounding soft tissue oedema that raise up the mass effect. MRI is better than CT due to its better soft tissues contrast [58]: involved anatomical regions and planes of the neck might be easier to recognize. In coronal planes, communication between mastoid and neck collections through the mastoid tip can be seen [59], thus helping the diagnosis of Bezold’s abscess. Notably, MRI does not use ionizing radiation and might be safer, especially when dealing with young/pediatric patients. MRI gadolinium-based contrast agents are also safer than CT iodine-based contrast agents in patients with impaired renal function (c) and in diabetic patients [60] and present less adverse effects. In the literature, MRI was performed in severe renal impairment (1/21) [61] or in patients of a young age (7/21 were pediatrics and additional 7/21 were younger than 35 years).

### 4.8. Treatment

If Bezold’s abscess is present or suspected, broad spectrum antibiotic therapy with good cerebrospinal fluid penetration should be started, and appropriate imaging performed to evaluate location and size of the abscess collection. The routinely instituted empirical broad-spectrum antibiotic therapy should cover most of the Gram-positive and Gram-negative aerobic and anaerobic pathogens, given the incidence of polymicrobial infections (19/67 cases among patients with referred microbiological cultures). Early surgery is often mandatory to establish drainage of the middle ear (also through a myringotomy) and mastoid cells. It is necessary to carry out a sampling of the purulent material. The results of pathogen tests allow to replace as soon as possible the initially administered, broad-spectrum antibiotic with one to which the pathogen has (or the pathogens have) a known susceptibility [62,63]. If a deep neck fluid collection exists concurrently with a coalescent mastoiditis, a post-auricular incision is made, and a complete mastoidectomy should be performed in addition to the drainage of the deep neck abscess via a trans-cervical approach. After surgical drainage of the deep neck abscess collection, contrast-enhanced imaging control is recommended at 48–72 h, prior to removal of the suction drains.

These observations seem to be in line with a recent study that focused its attention on the management of Bezold’s abscesses in a subgroup of cases of the last 20 years [64].

## 5. Conclusions

Bezold’s abscess entails a high risk for life-threatening intracranial, neck, and chest complications. The knowledge of some clinical, anatomical, diagnostic, and treatment aspects derived from literature and this comprehensive case analysis can support clinicians in the most appropriate management, especially considering the imaging features that can save time for a prompt diagnosis and treatment.

## Figures and Tables

**Table 1:** Results of 67 microbial cultures in Bezold’s abscess.

	Number of Patients (%)
Multimicrobial culture	19 (28%)
Inconclusive	14 (20%)
Unimicrobial culture	34 (51%)
Gram+	33 (49%)
Gram-	22 (33%)
Mycobacterium tuberculosis	3 (4%)
Fungus	1 (2%)

**Table 2:** Clinical presentation of Bezold’s abscess.

Signs and Symptoms	Number of Patients (%)
Neck pain/tenderness	57 (59%)
Neck swelling	60 (62%)
Fever	54 (56%)
Otorrhea	54 (56%)
Hearing loss	39 (40%)
Mixed	7 (18%)
Conductive	8 (21%)
Neurosensorial	3 (8%)
**Otitis**	**42 (43%)**
Acute	22 (23%)
Chronic	18 (19%)
Recurrent	2 (2%)
Otalgia	40 (41%)
Mastoid pain/tenderness	36 (37%)
Mastoid swelling	34 (35%)
Limitation in neck movements	25 (26%)
Neck erythema	23 (24%)
Neck stiffness/torticollis	12 (12%)
Headache	14 (14%)
**Cranial nerves paralysis**	
Facial nerve	8 (8%)
Hypoglossal nerve	1 (1%)
Abducens nerve	1 (1%)
**Abnormal tympanic membrane**	**42 (43%)**
Perforated	14 (33%)
Inflamed	10 (24%)
Retracted	7 (17%)
Bulging	5 (12%)
Thickened	5 (12%)
Dull	2 (5%)
**Clinical history**	
Cholesteatoma	39 (40%)
Diabetes	5 (5%)
Previous oto-mastoid surgery	11 (11%)

## Data Availability

The data presented in this study are available on request from the corresponding author.

## References

[1] Secko M., Aherne A. (2013). Diagnosis of Bezold Abscess Using Bedside Ultrasound. J. Emerg. Med..

[2] Lepore M.L., Hogan C.J., Geiger Z. (2020). Bezold Abscess. StatPearls.

[3] Bezold F. (1908). Text-Book of Otology for Physicians and Students: In 32 Lectures.

[4] Lovato A., de Filippis C. (2019). Bezold Abscess: A Rare Complication of Acute Otitis Media. Otol. Neurotol..

[5] Ren Y., Sethi R.K.V., Stankovic K.M. (2018). Acute Otitis Media and Associated Complications in United States Emergency Departments. Otol. Neurotol..

[6] Zer Toros S., Tepe Karaca C., Kalaycik Ertugay C., Senbayrak S., Ertugay O.C., Seneldir L. (2017). Simultaneous Coexistence of Complications of Chronic Otitis Media in the Same Case. J. Int. Adv. Otol..

[7] Nelson D., Jeanmonod R. (2013). Bezold Abscess: A Rare Complication of Mastoiditis. Am. J. Emerg. Med..

[8] Castillo M., Albernaz V.S., Mukherji S.K., Smith M.M., Weissman J.L. (1998). Imaging of Bezold’s Abscess. Am. J. Roentgenol..

[9] Hoshi H. (1962). Sex Difference in the Shape of the Mastoid Process in Norma Occipitalis and Its Importance to the Sex Determination of the Human Skull. Okajimas Folia Anat. Jpn..

[10] Kozerska M., Skrzat J., Szczepanek A. (2015). Application of the Temporal Bone for Sex Determination from the Skeletal Remains. Folia Med. Cracov..

[11] Wu J.-F., Jin Z., Yang J.-M., Liu Y.-H., Duan M.-L. (2012). Extracranial and Intracranial Complications of Otitis Media: 22-Year Clinical Experience and Analysis. Acta Otolaryngol..

[12] Mansour S., Magnan J., Nicolas K., Haidar H. (2018). Acute Otitis Media and Acute Coalescent Mastoiditis. Middle Ear Diseases.

[13] Nardis P.F., Bellelli A., D’Ottavi L.R. (1992). Cholesteatoma of the Prussak’s space. Diagnosis with computerized tomography. Radiol. Med..

[14] Smouha E.E., Levenson M.J., Anand V.K., Parisier S.C. (1989). Modern Presentations of Bezold’s Abscess. Arch. Otolaryngol.-Head Neck Surg..

[15] Minerva M., Valeggia S., Fusetti S., Zanoletti E., Manara R., Brotto D. (2021). Bezold’s Abscess Secondary to os Tympanicum Cholesteatoma in Goldenhar Syndrome. BJR Case Rep..

[16] Li L., Ren J. (2012). Aural Cholesteatoma with Upper Neck Extension. Auris. Nasus. Larynx.

[17] Hughes G.B., Damiani K.A., Kinney S.E., Levine H.L. (1980). Aural Cholesteatoma Presenting as a Large Neck Mass. Otolaryngol. Neck Surg..

[18] Al-Zahid S., Izadi D., Day C., Wilson A., Stone C., Smith J. (2019). A Novel Airway Management Strategy for Cervical Necrotising Fasciitis Secondary to Bezold’s Abscess. Ann. R. Coll. Surg. Engl..

[19] Arora V. (2015). Bezold′s Fistula: An Unusual Presentation of Cholesteatoma. Indian J. Otol..

[20] Kale P.C., Unnikrishnan A., Thomas J., Rajashekhar R.P., Karodpati N.S. (2016). Unusual Presentation Of Mastoid Abscess. J. Evid. Based Med. Healthc..

[21] Kanawaku Y., Yanase T., Hayashi K., Harada K., Kanetake J., Fukunaga T. (2013). An Autopsy Case of Otogenic Intracranial Abscess and Meningitis with Bezold’s Abscess: Evaluation of Inflammatory Bone Destruction by Postmortem Cone-Beam CT. Leg. Med..

[22] Leung V. (2016). Paediatric Acute Mastoiditis Complicated by Bezold Abscess and Epidural Empyema without Bone Erosion. https://www.eurorad.org/case/13567.

[23] Pradhananga R. (2014). An Unusual Complication of Chronic Suppurative Otitis Media: Bezold Abscess Progressing to Scapular Abscess. Int. Arch. Otorhinolaryngol..

[24] Scott S. (1935). An Uncommon Type of Bezold’s Mastoiditis. Proc. R. Soc. Med..

[25] Agrawal A., Singh H.P., Kumar D. (2011). Otogenic Anterior Chest Wall Abscess: A Rare and Unique Presentation of Bezold’s Abscess. Int. J. Otorhinolaryngol. Clin..

[26] Silva V.A.R., Almeida A.S., Lavinsky J., Pauna H.F., Castilho A.M., Chone C.T., Crespo A.N. (2020). Thorax Necrotizing Fasciitis Following Bezold’s Abscess. Clin. Case Rep..

[27] Suwita B.M., Suroyo I., Yunus R.E. (2020). Bezold’s Abscess with Intracranial Complications of Mastoiditis in an Immunocompetent Adult: A Rare Case. Otolaryngol. Case Rep..

[28] Moloy P.J. (1982). Anaerobic Mastoiditis: A Report of Two Cases with Complications. Laryngoscope.

[29] Pearson C.R., Riden D.K., Garth R.J.N., Thomas M.R. (1994). Two Cases of Lateral Sinus Thrombosis Presenting with Extracranial Head and Neck Abscesses. J. Laryngol. Otol..

[30] Nawas M.T., Daruwalla V.J., Spirer D., Micco A.G., Nemeth A.J. (2013). Complicated Necrotizing Otitis Externa. Am. J. Otolaryngol..

[31] Samuel J., Fernandes C.M.C. (1985). Otogenic Complications with an Intact Tympanic Membrane. Laryngoscope.

[32] Marioni G., de Filippis C., Tregnaghi A., Marchese-Ragona R., Staffieri A. (2001). Bezold’s Abscess in Children: Case Report and Review of the Literature. Int. J. Pediatr. Otorhinolaryngol..

[33] Angurana S., Bansal A., Mehta A., Bansal S., Jayashree M. (2019). Mastoiditis, Bezold Abscess, Dural Sinus Thrombosis, and Bilateral Abducens Nerve Palsy in a Child with Chronic Suppurative Otitis Media: A Rare Combination. Int. J. Pediatr..

[34] Bihani A., Dabholkar J., Dokhe Y., Hardikar P. (2016). Bezold’s Abscess Presenting as Parapharyngeal Abcessc. Otolaryngol. Online J..

[35] Effat K. (2014). Spontaneous Drainage of a Bezold Neck Abscess into the Middle-Ear Cleft: A Rare Incident. Egypt. J. Otolaryngol..

[36] Gaffney R.J., O’Dwyer T.P., Maguire A.J. (1991). Bezold’s Abscess. J. Laryngol. Otol..

[37] Janardhan N., Nara J., Peram I., Palukuri S., Chinta A., Satna K. (2012). Congenital Cholesteatoma of Temporal Bone with Bezold’s Abscess: Case Report. Indian J. Otolaryngol. Head Neck Surg..

[38] Moisa I.I., Danziger E.J., Brauer R.J. (1987). Subperiosteal and Bezold’s Abscesses Complicating Cholesteatoma: A Case Report. Otolaryngol. Neck Surg..

[39] Neto J.L., Saffer M., Rotta F.T., Arrarte J.L.F., Brinckmann C.A., Ferreira P. (1998). Lateral Sinus Thrombosis and Cervical Abscess Complicating Cholesteatoma in Children: Case Report and Review. Int. J. Pediatr. Otorhinolaryngol..

[40] Eswaran S., Kumar S., Kumar P. (2019). A Rare Case of Primary Tuberculous Otitis Media with Bezold’s Abscess. Indian J. Otolaryngol. Head Neck Surg..

[41] Patel K.M., Almutairi A., Mafee M.F. (2014). Acute Otomastoiditis and Its Complications: Role of Imaging. Oper. Tech. Otolaryngol.-Head Neck Surg..

[42] Lionello M., Manara R., Lora L., Mylonakis I., Fasanaro E., Torre B.L., Ottaviano G., Staffieri A., Ragona M. (2013). Case Report of Cholesteatoma Recurrence with Bezold’s Abscess Presenting as a Deep Neck Infection. B-ENT.

[43] Katayama K., Gomi H., Shirokawa T., Akizuki H., Kobayashi H. (2018). Bezold’s Abscess in a Diabetic Patient without Significant Clinical Symptoms. IDCases.

[44] Mantsopoulos K., Wurm J., Iro H., Zenk J. (2015). Role of Ultrasonography in the Detection of a Subperiosteal Abscess Secondary to Mastoiditis in Pediatric Patients. Ultrasound Med. Biol..

[45] Mądryz E.B., Leczycka M.W., Robert B., Krzeski A. (2018). Head and Neck Abscesses in Complicated Acute Otitis Media-Pathways and Classification. Otolaryngology.

[46] Rashid R.J., Naderpour M., Habibzadeh A., Jalili J., Mozayyan M. (2013). A Case of Bezold’s Abscess with an Unusual Extension to the Upper Thorax. J. Clin. Anal. Med..

[47] Radiopaedia.org. https://radiopaedia.org/articles/bezold-abscess?lang=us.

[48] Zapanta P.E., Chi D.H., Faust R.A. (2001). A Unique Case of Bezold’s Abscess Associated with Multiple Dural Sinus Thromboses. Laryngoscope.

[49] Elmubarak I., Ekezie J., Manwani S. (2021). Facial Nerve Palsy after Ear Infection: A Case Report. J. Sci. Innov. Med..

[50] Meissner H.C. (2011). What’s the Dx? Teen with Pain, Swelling, Discharge in Ear Canal. AAP News.

[51] Stokroos R. (2003). Radiology Quiz Case. Arch. Otolaryngol. Neck Surg..

[52] Malik K., Dever L.L., Kapila R. (2019). Bezold’s Abscess: A Rare Complication of Suppurative Mastoiditis. IDCases.

[53] McMullan B. (2009). Bezold’s Abscess: A Serious Complication of Otitis Media. J. Paediatr. Child Health.

[54] Saeedi M., Jahanshir A., Shirani F., Karimi E. (2016). Physical Examination Still Has the Leading Role: A Case of Bezold’s Abscess. J. Acad. Emerg. Med. Case Rep..

[55] Aikele P., Kittner T., Offergeld C., Kaftan H., Hüttenbrink K.-B., Laniado M. (2003). Diffusion-Weighted MR Imaging of Cholesteatoma in Pediatric and Adult Patients Who Have Undergone Middle Ear Surgery. Am. J. Roentgenol..

[56] Henninger B., Kremser C. (2017). Diffusion Weighted Imaging for the Detection and Evaluation of Cholesteatoma. World J. Radiol..

[57] Kálmán J., Horváth T., Liktor B., Dános K., Tamás L., Gődény M., Polony G. (2021). Limitations of non-echo planar diffusion weighted magnetic resonance imaging (non-EPI MRI) in cholesteatoma surveillance after ossicular chain reconstruction. A prospective study. Auris Nasus Larynx.

[58] Aulino J.M., Kirsch C.F.E., Burns J., Busse P.M., Chakraborty S., Choudhri A.F., Conley D.B., Jones C.U., Lee R.K., Luttrull M.D. (2019). ACR Appropriateness Criteria^®^ Neck Mass-Adenopathy. J. Am. Coll. Radiol..

[59] Uchida Y., Ueda H., Nakashima T. (2002). Bezold’s Abscess Arising with Recurrent Cholesteatoma 20 Years after the First Surgery: With a Review of the 18 Cases Published in Japan since 1960. Auris Nasus Larynx.

[60] ESUR.org. https://www.esur.org/fileadmin/content/2019/ESUR_Guidelines_10.0_Final_Version.pdf.

[61] Al-Baharna H., Al-Mubaireek H., Arora V. (2016). Bezold’s Abscess: A Case Report and Review of Cases over 14 Years. Indian J. Otol..

[62] Staffieri C., Fasanaro E., Favaretto N., La Torre F.B., Sanguin S., Giacomelli L., Marino F., Ottaviano G., Staffieri A., Marioni G. (2014). Multivariate Approach to Investigating Prognostic Factors in Deep Neck Infections. Eur. Arch. Otorhinolaryngol..

[63] Marioni G., Staffieri A., Parisi S., Marchese-Ragona R., Zuccon A., Staffieri C., Sari M., Speranzoni C., de Filippis C., Rinaldi R. (2010). Rational Diagnostic and Therapeutic Management of Deep Neck Infections: Analysis of 233 Consecutive Cases. Ann. Otol. Rhinol. Laryngol..

[64] Alkhaldi A.S., Alwabili M., Albilasi T., Almuhanna K. (2022). Bezold’s Abscess: A Case Report and Review of Cases over 20 Years. Cureus.

